# Executive Function and Academic Achievement in Primary School Children: The Use of Task-Related Processing Speed

**DOI:** 10.3389/fpsyg.2018.00582

**Published:** 2018-04-23

**Authors:** Rebecca Gordon, James H. Smith-Spark, Elizabeth J. Newton, Lucy A. Henry

**Affiliations:** ^1^Department of Psychology, Institute of Psychiatry, Psychology and Neuroscience, King's College London, London, United Kingdom; ^2^Division of Psychology, London South Bank University, London, United Kingdom; ^3^Division of Language & Communication Science, City, University of London, London, United Kingdom

**Keywords:** executive function, working memory, academic achievement, processing speed, updating, attention, inhibition, task-switching

## Overview

This article argues that individual differences in processing speed are important in the relationship between executive function (EF) and academic achievement in primary school children. It proposes that processing times within EF tasks can be used to predict academic attainment and aid in the development of intervention programmes.

## Executive function and academic achievement

Executive function (EF) is an umbrella term for a set of cognitive constructs required when routine behavior is insufficient to achieve a known goal; in such cases, executive control of attention is required (Norman and Shallice, 1980/1986). There is much evidence that this effortful attentional resource is limited (e.g., Schmeichel, 2007), and is used in prioritizing behavior, inhibiting irrelevant or inappropriate actions, maintaining information in short-term memory, filtering out irrelevant stimuli, and switching attention between tasks or rules (Diamond, 2006). Research generally considers task-switching, inhibition and updating^1^ to be the core EFs (e.g., Miyake et al., 2000). As such, studies investigating the structure of EFs in children have typically examined these three constructs (e.g., Huizinga et al., 2006; van der Ven et al., 2013).

Research over the past 20 years has shown that children exhibit developmental increases in EF from infancy to adulthood (Anderson, 2002) and that such increases are linked to academic achievement (e.g., Best et al., 2011). For example, studies have linked inhibition to mathematics (Bull and Scerif, 2001), task-switching to reading (e.g., van der Sluis et al., 2007) and mathematics (e.g., Bull and Scerif, 2001), and updating (Van der Ven et al., 2012) or working memory (WM) (Cragg et al., 2017) to mathematics. There is strong evidence to suggest that an understanding of how EF facilitates learning can enable early cognitive deficit identification and subsequent intervention programmes (e.g., Ribner et al., 2017).

## Variability in method and findings

Since the seminal work of Miyake et al. (2000), studies have increasingly looked to latent variable analysis to understand the structure and role of EFs. By calculating the variance shared between tasks purporting to measure a certain EF construct, a latent variable for that construct is created. However, there is variability in the findings from such studies. For example, studies have found a two-factor structure of WM and shifting, wherein inhibition was not identifiable in 7- to 21-year-olds (Huizinga et al., 2006) and 9- to 12-year-olds (van der Sluis et al., 2007). Conversely, other research found that WM and a combination of inhibition and shifting created a two-factor model in 6- to 8-year-olds (van der Ven et al., 2013) and 5- to 13-year-olds (Lee et al., 2013).

Further to this, there has also been concern regarding the reliability of EF measures (see Miyake and Friedman, 2012, for a review). Studies that have found EF to predict school achievement have varied in the methods employed. For example, studies linking inhibition to mathematics have used a single measure to represent this EF (e.g., Bull and Scerif, 2001) or have not used a specific measure of inhibition, but used inference from other assessments, such as the ability to reject irrelevant information in a WM task (e.g., Passolunghi et al., 1999).

To address these methodological issues, latent variable analysis has been used to examine the relationship between EF and academic abilities. When this has been done, a different story starts to emerge than that shown in earlier studies. In a study of 211 7- and 8-year-olds, Van der Ven et al. (2012) found that, after controlling for updating ability, latent constructs for inhibition and task-switching did not predict mathematical performance. Similarly, van der Sluis et al. (2007) examined the contributions of inhibition, task-switching and updating to reading, arithmetic and non-verbal reasoning in 9- to 12-year-olds. No latent inhibition factor was identified, and the task-switching factor predicted only non-verbal reasoning and reading performance. However, updating related to reading, arithmetic, and non-verbal reasoning. In fact, when updating was included as a predictor in such studies, the variance in academic ability explained by inhibition and task-switching was usually no longer significant (see also Toll et al., 2011).

These studies illustrate that there remain unanswered questions regarding the structure of EF, and its relationship with academic attainment. In this article, it is argued that considering the role of processing speed in EF task performance may assist in answering these questions. The basis for this argument lies in the findings of the following studies that investigated issues in EF measurement.

## Addressing issues in EF measurement

Processing speed has been shown to influence the structure of EF. For example, van der Ven et al. (2013) controlled for baseline speed in measures of EF, and used speed scores to indicate inhibition and shifting ability. On the basis of their findings, they argued that variations in the structural organization of EF might be the result of differences in the methodologies used (i.e., controlling or not controlling for speed). Further evidence supports this finding. Huizinga et al. (2006) could not identify an inhibition factor in 9- to 12-year-olds, when controlling for processing speed. In addition, McAuley and White (2011) found that processing speed accounted for significant variance in the developmental trajectory of WM and inhibition. Acknowledging some degree of speculation, they suggested that processing speed may enable faster interpretation of environmental cues which indicate the suitability of certain purposeful behaviors. These studies provide evidence that processing speed is important in EF and are consistent with Fry and Hale (1996, 2000) who argued that processing speed underpins all EF constructs.

Given the varied findings regarding the link between EF structure and academic ability, there is value in investigating how processing speed may influence this relationship. Although studies have investigated this, they have used speeded tasks that sit outside of EF tasks (e.g., Bayliss et al., 2005; Berg, 2008; Passolunghi and Lanfranchi, 2012). However, van der Sluis et al. (2007) looked at the role of processing speed within EF, and how EF then relates to academic ability. They did this to address an important issue in EF measurement, the task impurity problem. This problem arises due to the need for participants to engage other, non-executive, cognitive abilities when completing EF tasks (Burgess, 1997). van der Sluis examined the structure of EF and its relationship to reading, arithmetic and non-verbal reasoning in 9- to 12-year-olds. Seven tasks were used and performance on each was separated into executive and non-executive components. For example, a non-executive component required rapid naming of a letter and the executive component required naming of the letter dependent on its location within a square. Performance on the simple processing component of the task was separated from performance when there was an executive load. The two performance indices (i.e., accuracy in the executive component and processing time in the non-executive component) were used to predict academic ability. A shifting and an updating factor were identified when controlling for the variance explained by the speeded non-executive task and updating was linked to reading and mathematics. However, performance on the non-executive speeded components was more strongly related to arithmetic and reading ability than the executive-loaded components.

So far we have discussed the evidence that processing speed is important in EF structure, and that it influences how EF constructs relate to academic achievement. This article now argues that identifying individual differences in processing speeds when there is an executive load can explain the link between EF and academic attainment.

## The use of task-related processing speed to predict academic achievement

There is considerable evidence for links between processing speed, EF and academic achievement. This is, in part, evident in the similar developmental trajectories of the three abilities. Information processing has been shown to develop rapidly from 3 to 5 years of age (Espy et al., 2006), with significant improvements observed in 9- and 10-year-olds (Kail, 1986). This trend is commensurate with the developmental increases in EF (Anderson, 2002; Demetriou et al., 2014) and academic achievement (Best et al., 2011; Demetriou et al., 2014) mentioned previously. Links between processing speed and EF are further supported by early research explaining capacity increases in WM. According to the task-switching (Towse and Hitch, 1995) and resource sharing (Daneman and Carpenter, 1980) hypotheses, a developmental increase in processing speed can explain an enhanced ability to refresh decaying memory items (Towse and Hitch, 1995) or free up storage space (Daneman and Carpenter, 1980). In addition, Bayliss et al. (2005) found that processing speed contributed, in part, to developmental improvements in complex span task performance due to decay prevention and faster reactivation of memory items. Furthermore, the time-based resource-sharing model of WM (Camos and Barrouillet, 2011) argues for the development of an attentional switching capability to explain increases in WM capacity at approximately 7 years of age; and this ability is demonstrated by a linear relationship between processing speed and storage capacity in complex span tasks.

Even when examining WM alone, placing stress on a participant's ability to process information more quickly has resulted in stronger relationships with measures of reading, mathematics and non-verbal reasoning (Lépine et al., 2005). Lépine and colleagues restricted the time available for participants to process stimuli in complex span tasks, before asking them to recall the memoranda related to the task. When comparing performance to that on tasks with no time restrictions, it was found that time-restricted tasks showed stronger links to performance on the measures of reading, mathematics and non-verbal reasoning.

The research discussed in this article provides evidence that individual differences in EF may be underpinned by the speed with which information can be processed when there are executive demands. Furthermore, the relationship between EF and academic abilities is strengthened when time restrictions are placed on the processing component of EF tasks (Lépine et al., 2005). This suggests that individual differences in processing speed during executive control of attention may explain differences in EF, and its relationship with academic achievement. However, the required evidence may only be identifiable if studies unpack the tasks used to measure EF in order to identify underlying mechanisms. Some earlier studies have been successful in adopting this approach, whereby the components of EF measures are extracted and analyzed as predictors of academic abilities. For example, the time taken to recall memoranda in complex span tasks designed to assess WM capacity have been shown to predict reading ability (Cowan et al., 2003; Towse et al., 2008).

## Application

The purpose of identifying which cognitive constructs influence academic achievement is to enable subsequent intervention programmes (e.g., Ribner et al., 2017). As evidence suggests that processing speed as early as 5 months of age influences long-term EF (see Cuevas and Bell, 2014), it is unlikely that intervention programmes aimed at improving processing speed in primary school would be beneficial. However, as lesson structures in UK primary schools are time-restricted, often to 20-min slots (Qualifications and Curriculum Authority, 2002), this may hinder children with slower processing speeds. If there is a greater awareness of the role of processing speed in tasks that rely on EF, then school intervention programmes can make reasonable time adjustments for children who struggle due to a deficit in this area; similar to interventions which exist for developmental disorders such as dyslexia.

## Summary

The evidence discussed here provides opportunities to develop a new approach to examining the relationship between EF and academic achievement. Future studies should clarify the role of executive-loaded processing speed in tasks by measuring individual differences in processing times. Using these as predictors of academic attainment, may allow identification of children who, due to slower processing speeds, struggle with academic tasks when there is an executive load.

## Author contributions

RG: Conception of article and drafting of manuscript; RG and JS-S: Critical revision of the text; EN and LH: Review of text; RG, JS-S, EN, and LH: Approved the final version of the manuscript.

### Conflict of interest statement

The authors declare that the research was conducted in the absence of any commercial or financial relationships that could be construed as a potential conflict of interest. The reviewer, LV, and handling Editor declared their shared affiliation.

## Footnotes

^1^Updating has been defined as the cognitive ability to store, monitor and modify information in an accessible state (e.g., Miyake et al., 2000). St Clair-Thompson and Gathercole (2006) assessed children on four WM tasks and two updating measures. It was found that all the tasks loaded together on the same factor. They concluded that measures of WM and updating assess the same underlying construct. Updating in this article is thus considered synonymous with WM.
